# Molecular Weapons Contribute to Intracellular Rhizobia Accommodation Within Legume Host Cell

**DOI:** 10.3389/fpls.2019.01496

**Published:** 2019-11-26

**Authors:** Camille Syska, Renaud Brouquisse, Geneviève Alloing, Nicolas Pauly, Pierre Frendo, Marc Bosseno, Laurence Dupont, Alexandre Boscari

**Affiliations:** ^1^Université Côte d’Azur, INRA, CNRS, ISA, Sophia Antipolis, France; ^2^Laboratoire des Interactions Plantes-Microorganismes, INRA, CNRS, Université de Toulouse, Castanet-Tolosan, France

**Keywords:** legumes, symbiosis, bacteroid, reactive oxygen species, nitric oxide, nitrogen- fixation, nodule-specific cysteine rich peptides, toxin–antitoxin

## Abstract

The interaction between legumes and bacteria of rhizobia type results in a beneficial symbiotic relationship characterized by the formation of new root organs, called nodules. Within these nodules the bacteria, released in plant cells, differentiate into bacteroids and fix atmospheric nitrogen through the nitrogenase activity. This mutualistic interaction has evolved sophisticated signaling networks to allow rhizobia entry, colonization, bacteroid differentiation and persistence in nodules. Nodule cysteine rich (NCR) peptides, reactive oxygen species (ROS), reactive nitrogen species (RNS), and toxin–antitoxin (TA) modules produced by the host plants or bacterial microsymbionts have a major role in the control of the symbiotic interaction. These molecules described as weapons in pathogenic interactions have evolved to participate to the intracellular bacteroid accommodation by escaping control of plant innate immunity and adapt the functioning of the nitrogen-fixation to environmental signalling cues.

## Introduction

The nitrogen-fixing symbiosis (NFS) results from the relationship between plants of the legume family and soil bacteria referred to as rhizobia. After a recognition step, bacteria infect legume roots, induce the formation of specialized root organs, the nodules, and colonize nodule cells by endocytosis to form structures called symbiosomes (Ferguson et al., 2010). Inside symbiosomes, bacteria differentiate into bacteroids that can convert atmospheric dinitrogen (N_2_) into ammonia (NH_4_^+^), *via* the nitrogenase activity. NH_4_^+^ is then transferred to the whole plant through either amino acids or ureide compounds (Oldroyd and Downie, 2008; Masson-Boivin et al., 2009). NFS provides substantial agronomic and environmental benefits such as the substitution to nitrogen (N) fertilizer inputs to increase the plant yields (Vitousek et al., 2013).

The setting of NFS depends on a signal exchange. An initial plant defense response is observed during the first hours of the interaction with the rhizobium, and then is actively suppressed after the recognition. How rhizobia are recognized as symbionts rather than pathogens by the host plant is well described (Jones et al., 2007; Soto et al., 2009), and the strategies of the plants to adjust their own defense systems to enable rhizobia entry, colonization, and differentiation are detailed in several reviews (Oldroyd et al., 2011; Oldroyd, 2013). Recent reports support the hypothesis that the regulation of immune response does not end at the recognition stage, but rather continue to allow rhizobial long-term accommodation inside the plant cells (Cao et al., 2017; Zipfel and Oldroyd, 2017; Berrabah et al., 2018; Yu et al., 2019). Multiple compounds such as nodule-specific cysteine rich (NCR) peptides, reactive oxygen species (ROS), reactive nitrogen species (RNS) and toxin–antitoxin (TA) modules have been shown to control the setup and the functioning of the interaction between the two partners The purpose of the present review is to provide an overview of the role of these compounds, described as weapons in pathogenic interactions, in the intracellular bacteroid accommodation (rhizobial colonization, differentiation, and control of plant innate immunity for persistence) and the adjustment of the nitrogen-fixation activity to environmental signalling cues.

### Reactive Oxygen Species (ROS) in Bacterial Colonization of the Plant Cell and Bacteroid Persistence in the Nodule

ROS are involved in adaptation to environmental perturbations (Apel and Hirt, 2004; Waszczak et al., 2018). They are also essential for promoting normal cellular processes in bacteria and plants (Mittler, 2017). The level of ROS in cells depends on the tight regulation of a complex array of ROS generating systems and detoxification mechanisms, and antioxidant metabolites like glutathione and ascorbate. The balance between ROS production and detoxification regulates the cellular redox homeostasis in plants as well as in bacteria (Apel and Hirt, 2004).

**Figure 1:**
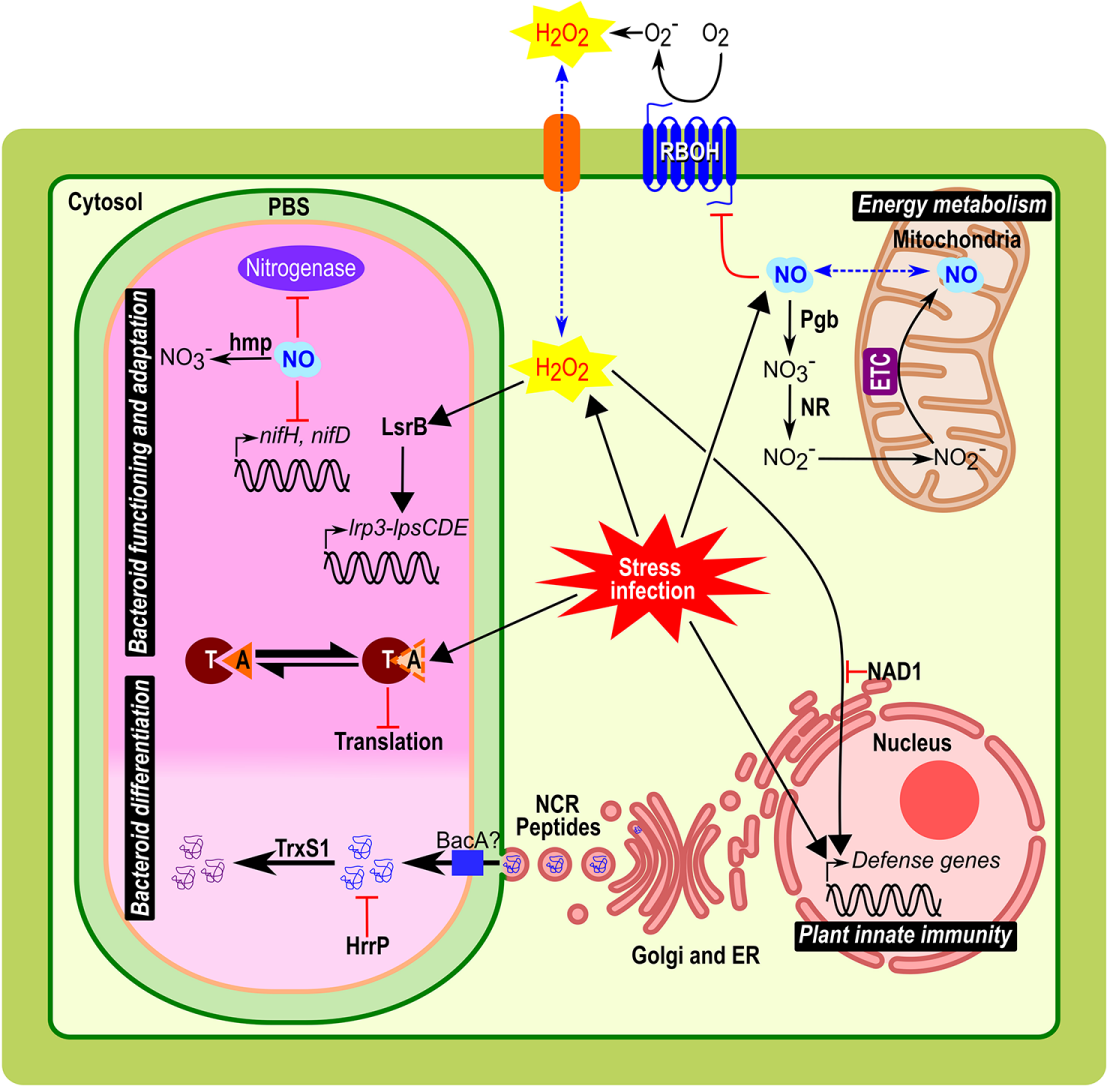
Implication and connection of ROS, NO, NCR peptides, and TA modules in symbiosomes from *Medicago* root nodules. Biological role of these compounds during bacteroid differentiation, nodule functioning and adaptation, plant innate immunity, and energy metabolism are represented. Plant host cells infected by bacteria/bacteroid, implied various stress responses such as oxidative/nitrosative stress, acidic pH, microoxia, and exposure to NCRs. In the symbiosome, the clear part corresponds to the infection zone and the dark pink to the fixation zone with bacteria differentiated in bacteroid. Black arrows indicate metabolism reaction or downstream signal transduction pathways; red arrows indicate regulation mechanism (activation with arrowhead or repression with bar-headed lines). Blue dotted arrow indicates a diffusion through the membrane. Abbreviations: PBS, peribacteroid space; NR, nitrate reductase; Pgb, Phytoglobin; RBOH, respiratory burst oxidase homologs; O_2_^−^, superoxide radical; H_2_O_2_, hydrogen peroxide; ETC, electron transfer chain; NO, nitric oxide; ER; endoplasmic reticulum; NCR peptides, nodule-specific cysteine-rich peptides; Hmp, flavohemoglobin; NAD1, Nodules with Activated Defence 1; TrxS1, Thioredoxine S1; HrrP, Host-range restriction peptidase; LsrB, LysR transcription factor; T, toxin; A, antitoxin.

In plants, the respiratory burst oxidase homologs (RBOH) proteins (also called NADPH oxidase) emerged as the major sources of apoplastic ROS (**Figure 1**) and key players in the redox signaling during pathogen infection and other processes (Kadota et al., 2015; Liu and He, 2016; Montiel et al., 2016). Some members of this multigenic family are differentially expressed in *Medicago truncatula* nodule tissues and play different roles from the establishment of the symbiotic interaction to the functioning of mature nodule (Marino et al., 2011; Montiel et al., 2018). The reduction in the N_2_-fixation capacity in transgenic roots knocked-down for *MtRbohA* was the first evidence of a RBOH involvement in nodule functioning (Marino et al., 2011)(**Table 1**). Authors suggested that MtRBOHA activity contributes to the communication between the plant and the microsymbiont. Hydrogen peroxide production was visualized in *M. truncatula* infection zone and regulates genes involved in the nodulation process (Andrio et al., 2013). Similar results were obtain in *Phaseolus vulgaris* using knocked-down *PvRbohA* gene (Arthikala et al., 2017). Moreover, the roots overexpression of *PvRbohB* increases the number of bacteroids in the symbiosomes and improves biological N_2_-fixation in *P. vulgaris* (Arthikala et al., 2014). In contrast, mutations of *NAD1* gene (Nodules with Activated Defence) in *M. truncatula* activate a strong defence response after rhizobia are released from infection threads into plant cells, leading to necrotic cell death of symbiotic cells (Wang et al., 2016). The knock-out of either *MtRbohB*, *MtRbohC*, or *MtRbohD* in the *nad1* mutant reverts this cell death phenotype indicating that nodule innate immunity is notably mediated by RBOH activity (Yu et al., 2018; Yu et al., 2019). These data provide evidences that MtRBOH-mediated ROS production has positive and negative functions in the reception of the microsymbionts in the nodule cells.

**Table 1:** Non-exhaustive summary of genes involved in ROS, NCR, NO, and TA modules pathways within legume nodule cells.

	Mutant/transgenic line	Origin	Proteic activity	Symbiotic function		Reference
ROS	*nad1*	*M. truncatula*	Nodule activated defense protein -uncharacterized		Nodule innate immunity, Bacteroid differenciation/survival, N_2_-fixation	Wang et al. (2016)
	RbohA: RNAi				N_2_-fixation	Marino et al. (2011)
	RbohA: RNAi	*P. vulgaris*	Respiratory burst oxidase homolog - ROS production		Bacterial infection, Nodule formation, Bacteroid survival, N_2_-fixation	Arthikala et al. (2017)
	RbohB: RNAi				Bacterial infection, Nodule formation, N_2_-fixation	Montiel et al. (2012)
	RbohB: OE				Bacterial infection, Nodule formation, Bacteroid differenciation, N_2_-fixation	Arthikala et al. (2014)
	*gshB*	*S. meliloti*	ROS detoxification enzymes		Nodule formation, N_2_-fixation	Harrison et al. (2005)
	*trxL*				N_2_-fixation	Castro-Sowinski et al. (2007)
	*grx1*				N_2_-fixation	Benyamina et al. (2013)
	*grx2*				Nodule formation, N_2_-fixation	
	*sodA*				N_2_-fixation, Bacteroid differenciation	Santos et al. (2000)
	*katA/katC; katB/katC*				Nodule formation, Infection, N_2_-fixation, Bacteroid differenciation	Jamet et al. (2003)
	*lsrB*		LysR transcription factor		Infection, Bacteroid differenciation/survival, N_2_-fixation	Luo et al. (2005); Tang et al. (2013); Tang et al., (2014); Tang et al., (2017)
NCR	*dnf7-2* deletion mutant	*M. truncatula*	Antimicrobial peptide NCR169		Bacteroid survival/persistence	Horváth et al. (2015)
	*dnf4* deletion mutant	*M. truncatula*	Antimicrobial peptide NCR211- Symbiont Specificity		Bacteroid survival/persistence	Kim et al. (2015)
	*NFS1-/- (NCR*α-β)	*M. truncatula*	Antimicrobial peptide - Symbiont Specificity		Bacteroid survival, Senescence	Yang et al. (2017)
	Trx s1: RNAi	*M. truncatula*	Thioredoxin-NCR reduction		Bacteroid differenciation	Ribeiro et al. (2017)
	Trx s1: OE					
	*bacA*	*S. meliloti*	ABC transporter- Symbiont protection against NCRs		Bacteroid differenciation	Haag et al. (2011)
	*bclA*	*B. japonicum*			Bacteroid differenciation/survival	Guefrachi et al. (2015)
	*hrrP*	*S. meliloti*	M16A family metallopeptidase- Escape NCR control		Bacteroid fitness	Price et al. (2015)
NO	Hb1: RNAi	*L. japonicus*	Leghemoglobin- degradation of nitric oxide		N_2_-fixation	Ott et al. (2005)
	Hb1: OE		Phytoglobin- degradation of nitric oxide		N_2_-fixation	Shimoda et al. (2009)
	Hb1: OE	*A. firma*				
	*hmp*	*S. meliloti*	Flavohemoprotein- NO degradation		Bacteroid survival, N_2_-fixation, Senesence	Cam et al. (2012); Meilhoc et al. (2013); Blanquet et al. (2015)
	*hmp++*				
	*norB*					Blanquet et al. (2015)
	*nnrS1*					
TA modules	*vapC-4* (*ntrR*)	*S. meliloti*	VapB (antidote), VapC (site-specific RNase)		Nodule formation	Dusha et al. (1989)
					N_2_-fixation, Senescence	Oláh et al. (2001)
	*vapB-5*				Nodule formation, Bacteroid differenciation	Lipuma et al. (2014)
	*vapC-5*				N_2_-fixation, Bacteroid survival, Senescence	
	*bat/bto = vapBC*	*B. japonicum*			Nodule formation, N_2_-fixation	Miclea et al. (2010)

Genes studied have a rhizobial (orange) or a plant (light green) origin. The nitrogen-fixing phenotype of the mutant or transgenic line is depicted in green if defective or in pink if improved in the column symbiotic function.

To cope with the plant ROS production, the microsymbiont contains a number of antioxidants and ROS-scavenging enzymes to preserve the bacteroids against ROS damages (Puppo et al., 2005; Becana et al., 2010). Analysis of bacterial mutants deficient in glutathione synthetase (*gshB*), thioredoxin (*trxL*), glutaredoxins (*grx1*, or *grx2*), superoxide dismutase (*sodA*), and catalases (double mutants *katA/katC* or *katB/katC*) showed that the alteration in antioxidant pools as well as the mutation of ROS detoxification enzymes impact the formation of nodules, decrease the N_2_-fixing capacity and induce a premature nodule senescence (**Table 1**)(Santos et al., 2000; Jamet et al., 2003; Harrison et al., 2005; Castro-Sowinski et al., 2007; Benyamina et al., 2013). Besides, nodules induced by *a Sinorhizobium meliloti* deletion mutant of *lsrB*, which encodes a LysR transcription factor acting as a ROS regulator, showed premature senescence with impaired bacteroid differentiation (Luo et al., 2005; Tang et al., 2013). LsrB was found to induce the expression of the *lrp3*-*lpsCDE* operon involved in lipopolysaccharide biosynthesis required for infection or bacteroid survival in host cells (**Figure 1**)(Tang et al., 2014) and that of γ-glutamylcysteine synthetase, involved in glutathione synthesis (Tang et al., 2017).

### Nodule-Specific Cysteine Rich (NCR) Peptides and Terminal Bacteroid Differentiation

NCR peptides have been specifically found in the Inverted Repeat-Lacking Clade (IRLC) legumes such as *Medicago* spp., and in Dalbergoid legumes such as *Aeschynomene* spp., where bacteria are terminally differentiated to polyploïd non-dividing bacteroids (Mergaert et al., 2003; Mergaert et al., 2006; Alunni and Gourion, 2016). They encode highly divergent peptides, which resemble defensin-type antimicrobial peptides involved in plant and animal innate immunity (Mergaert et al., 2003). Indeed, some NCR peptides have a strong *in vitro* antimicrobial activity when applied to free-living bacteria (Van de Velde et al., 2010; Maróti and Kondorosi, 2014; Farkas et al., 2017).

Almost all NCR genes are exclusively expressed in the infected cells of nodules and their products are targeted to the symbiosome through the endoplasmic reticulum secretory system (**Figure 1**)(Wang et al., 2010; Guefrachi et al., 2014). Challenge of cultured bacteria with synthetic NCR peptides and ectopic expression of NCR peptides in legumes devoid of NCR genes cause features of bacteroid differentiation, demonstrating that these NCR peptides are sufficient to induce the irreversible differentiation (Van de Velde et al., 2010). The number of NCR genes is remarkably variable (from 7 in *Glycyrrhiza uralensis* to over 700 members in *M. truncatula*), and a positive correlation was found between the size of the NCR peptide family in the plant genome and the degree of bacteroid elongation (Montiel et al., 2017). Despite the large size of NCR peptide family in *M. truncatula* suggesting an extensive redundancy, NCR169, NCR211, and NFS1 are essential and the corresponding plant mutants are unable to establish a functional NFS (**Table 1**) (Horváth et al., 2015; Kim et al., 2015; Yang et al., 2017). Both NFS1 and NCR211 exemplify NCR peptides that control the survival of fully differentiated bacteroids instead of triggering the terminal differentiation of bacteroids. NFS1 controls the discrimination mechanisms against incompatible microsymbionts (Yang et al., 2017), provoking bacterial cell death and early nodule senescence in an allele-specific and rhizobial strain-specific manner, while NCR211 is required for bacteroid persistence inside symbiotic cells (Kim et al., 2015).

To survive exposure to toxic NCR peptides *S. meliloti* requires the integrity of the BacA ABC-transporter. A *bacA* mutant strain is unable to differentiate and rapidly dies after its release from infection threads (**Figure 1**) (Haag et al., 2011). Similarly, the BacA-like peptide transporter BclA of *Bradyrhizobium japonicum* is essential for bacteroid differentiation and survival in *Aeschynomene* nodule, which suggests that the NCR peptides uptake may be a common mechanism used by different rhizobia to counteract the toxic effect of peptides (Guefrachi et al., 2015). In the symbiosis between *Sinorhizobium freedi* and *G. uralensis* alternatively, bacteroid differentiation occurs *via* a *bacA*-independent pathway and is rather associated with LPS modification of the bacteroid outer membrane (Crespo-Rivas et al., 2016). Additionally, a *S. meliloti* natural strain can escape the control of NCR peptides and proliferate in nodules using the plasmid encoded host-range restriction peptidase Hrrp, which is able to digest NCR peptides *in vitro* (**Figure 1** and **Table 1**)(Price et al., 2015). The expression of *hrrp* increases the fitness of rhizobial strains while inhibiting N_2_-fixation in some plant ecotypes (Price et al., 2015).

Another layer of regulation may come from posttranslational modifications of NCR peptides (Marx et al., 2016). In particular, a nodule-specific thioredoxin, TrxS1, capable to reduce NCR peptides and targeted to symbiosomes, has been shown to be required for bacteroid differentiation, suggesting that NCR redox state is important *in planta* (**Figure 1** and **Table 1**)(Ribeiro et al., 2017). In this context, the redox control of the bacteroid differentiation probably occurs through the NCR peptide activity suggesting a crosstalk between the different regulators described in this review.

Considered together, these data indicate that the symbiosis efficiency of terminally differentiated bacteria is the outcome of a tight balance between the effects of NCR peptides and the ability of rhizobia to resist them. The rupture of this balance can lead to the activation of the plant innate immunity (Yu et al., 2019).

### Nitric Oxide (NO) in Functional Nodules: Microoxia, Energetic Metabolism

NO production was observed in functional nodules of *Lotus japonicus* and *M. truncatula*, mainly in the N_2_-fixation zone (Baudouin et al., 2006; Shimoda et al., 2009), and in the nodule senescence zone (Cam et al., 2012; Fukudome et al., 2018). Although the origin and the biological significance of NO production in nodules has been thoroughly analyzed over the last few years (Boscari et al., 2013; Hichri et al., 2015; Berger et al., 2019), there are still many questions to be clarified concerning the relative importance of the signaling/metabolic functions of NO versus its toxic action on host plant and symbiont.

Functional nodules are characterized by a microoxic environment to protect the bacterial nitrogenase from irreversible denaturation by oxygen (O_2_) which requires the setup of an O_2_ barrier in the outer cell layers of the nodule and the synthesis of leghemoglobin (Lb) (Appleby, 1992). In plant roots submitted to hypoxia, a “Phytoglobin-NO respiration” has been shown to use nitrite as a final electron acceptor instead of O_2_ to be reduced to NO by the mitochondrial electron transfer chain (ETC), which allows cell energy status retention (**Figure 1**) (Igamberdiev and Hill, 2009; Gupta and Igamberdiev, 2011). Accumulated data support the existence of such a Phytoglobin-NO respiration in *M. truncatula* and *Medicago sativa* nodules, in which both nitrate reductase (NR) and ETC are involved in NO production and in the maintenance of the nodule energy state (Horchani et al., 2011; Berger et al., 2018).

Despite its role in acclimation to microoxic environment, NO is also a potent inhibitor of nitrogenase activity (Trinchant and Rigaud, 1982; Sasakura et al., 2006; Kato et al., 2010). In nodules of soybean plants subjected to flooding, the increase in NO production is associated with the repression of bacterial *nifH* and *nifD* (**Figure 1**), and this inhibition is partially reversed by the application of the NO scavenger cPTIO, which illustrates the inhibitory role of NO on the expression of nitrogenase genes (Sánchez et al., 2010). Furthermore, using both pharmacological approach, with NO-donors and scavengers, and molecular approach with transgenic plants with modified NO levels, several studies report that NO inhibits *in vivo* N_2_-fixing activity in soybean, *L. japonicus*, and *M. truncatula* nodules (**Table 1**)(Shimoda et al., 2009; Kato et al., 2010; Cam et al., 2012).

The biological activity of NO is mediated through redox-dependent protein modifications such as metal-nitrosylation, S-nitrosation and Tyr-nitration (Stamler et al., 2001; Besson-Bard et al., 2008). In *M. truncatula* mature nodules, 80 proteins have been reported to be S-nitrosated, most of them involved in primary metabolism, energy regeneration and nitrogen assimilation (Puppo et al., 2013). In this context, *M. truncatula* glutathione peroxidase 1 and glutamine synthetase 1a were shown to be regulated by NO through S-nitrosation and Tyr-nitration modifications (Melo et al., 2011; Castella et al., 2017).

Beside the nodule metabolism regulation, a participation of NO to the life-time of the symbiotic interaction was also observed (Cam et al., 2012). Increased NO level in nodule obtained either by using *S. meliloti* mutant strains deficient in the degradation of NO (*hmp*, *norB*, *nnrS1*)(**Table 1**), or by treating nodules with NO donors (Cam et al., 2012; Meilhoc et al., 2013; Blanquet et al., 2015) leads to premature nodule senescence. Conversely, by using *S. meliloti* mutant strains that over-expressed *hmp*, a decrease in NO level was observed correlated to a delay of nodule senescence (**Table 1**)(Cam et al., 2012). Therefore, NO concentration should be tightly controlled, in time and space, in both partners to avoid its toxic effects and to fulfil its signaling and metabolic functions during nodule functioning and under environmental stresses (Berger et al., 2019).

### Toxin-Antitoxin (TA) Systems in Bacteroid Adaptation in Infected Plant Cells

TA systems are key players of intracellular survival of invading bacteria during eukaryote interactions (Lobato-Márquez et al., 2016). TA genes encode a stable toxin and its cognate antitoxin. Depending on the antitoxin nature (RNA or protein) and its mode of action, TA modules are classified into six different types (I–VI). The type II, where both toxin and antitoxin are small proteins forming a stable complex, is the most abundant type in pathogens, particularly exposed to diverse micro-environments during host interaction (Ramage et al., 2009; Georgiades and Raoult, 2011). Due to the self-poisoning effect of the toxin, TA modules could be considered as intracellular molecular timebombs. TA expression is tightly regulated to allow either growth arrest and bacterial adaptation or cell death (Hayes and Kędzierska, 2014). Under various stress conditions, the antitoxin is degraded by bacterial proteases leading to the deregulation of the TA operon and delivery of the toxin which targets specific cellular functions (DNA replication, translation) (Gerdes et al., 2005). In phytopathogenic bacteria, TA have been recently demonstrated as involved in virulence and biofilm formation during plant infection (Shidore and Triplett, 2017; Martins et al., 2018).

Among the 29 chromosomal type II TA systems of *S. meliloti*, eleven belong to the VapBC family; VapB being the antitoxin and VapC the toxin, acting as a site-specific RNase (**Table 1**). The importance of two *vapBC* operons, *vapBC-4* (*ntrPR*) and *vapBC-5*, has been shown in *S. meliloti* during symbiotic interaction with *Medicago* sp. (Dusha et al., 1989; Lipuma et al., 2014). NtrPR was identified on the capacity of the toxin *ntrR* mutant (for nitrogen regulator) to form more nodules on alfalfa roots in the presence of exogenous ammonium (Dusha et al., 1989). This suggests that NtrR toxin is involved in the nodulation efficiency depending on the level of nitrogen supply. This module plays also a role in mature nodules in a nitrogen-tolerant manner, as *ntrR*-induced nodules have an enhanced N_2_-fixation capacity and an increased plant yield (Oláh et al., 2001). Regarding VapBC-5 module, the *vapC-5* toxin mutant improves the symbiotic interaction with alfalfa (increase in N_2_-fixation capacity and plant yield) associated to a delay in nodule senescence (Lipuma et al., 2014).

These *vapC* mutants have no free-living phenotypes. Therefore, TA modules might play a role in the bacterial adaptation to infection stresses (metabolic shifting, acidic pH, microoxia, ROS, antimicrobial peptides, stresses known to activate pathogen TA modules (Lobato-Márquez et al., 2016)) (**Figure 1**). Thus, in a wild-type context, NtrPR and VapBC-5 modules likely limit the symbiotic interaction upon specific plant signals and/or contribute to the nodule senescence onset. The high number of TA systems in *S. meliloti* genome could be due to functional redundancy or to different roles independent of the NFS. Indeed, Milunovic et al. (2014) showed that the deletion of four TA operons from the pSyma and pSymb plasmids induces a cell toxicity phenotype in free living, with no symbiotic effect during alfalfa interaction (Milunovic et al., 2014). In contrast, in *B. japonicum* USDA110, the complete deletion of the *bat/bto* TA resulted in a limited production of soybean nodules associated to a reduced plant yield (Miclea et al., 2010). Such a phenotype suggests that this system might play a positive role on the symbiotic interaction with soybean, although this could also be linked to the pleiotropic effects observed for this deletion mutant in free-living conditions.

### Concluding Remarks

The evidences presented in this review show the importance of ROS, NO, NCR peptides, and TA modules in the intracellular bacteroid accommodation and the N_2_-fixation activity regulation. These molecules, considered in certain situations as cellular weapons, are necessary not only in the nodule functioning, but also in the rupture of the symbiosis under unfavourable conditions such as deficient bacterial symbionts, adverse environmental conditions or cellular aging. The importance of these regulatory elements is now clearly demonstrated, but their mode of action still remains to be fully deciphered. Identification of the molecular pathways involved in the regulation of the bacterial intracellular life during NFS will be helpful to dissect the crosstalk between these different regulatory elements. Evidences exist of the connection between ROS, NO, and NCR in plant cells to balance the plant immune response, to regulate the rhizobial differentiation and control the switch from bacteroid persistence to cell death. Among these recent findings it can be noted the involvement of three RBOH in the activation of immunity in *Medicago* nodules and the regulation of bacteroid differentiation *via* TrxS1-dependent redox regulation of some NCRs *in planta* (Ribeiro et al., 2017)(**Figure 1**). Furthermore, it was previously shown that NO could inhibit NADPH oxidase activity by post-translational modification (**Figure 1**)(Yun et al., 2011).

Similarly, the connection between TA, NO, ROS, and NCR produced by both partners represents a field of future interest to identify the signals involved in TA activation. The delayed senescence of nodules induced by the *vapC-5* toxin mutants conducted to higher the expression of NCR001 gene compared to control *Rhizobium* strain (Lipuma et al., 2014). Finally, a better understanding of these regulatory processes may give promising strategies to improve the NFS and reduce the use of fertilizers.

## Author Contributions

CS and AB conceived the idea of the review. All the authors were involved in the manuscript writing.

## Funding

This work was supported by the “Institut National de la Recherche Agronomique,” the “Centre National de la Recherche Scientifique,” the University of Nice-Sophia-Antipolis, and the French Government (National Research Agency, ANR) through the LABEX SIGNALIFE program (reference # ANR-11-LABX-0028-01) and the STAYPINK project (ANR-15-CE20-0005).

## Conflict of Interest

The authors declare that the research was conducted in the absence of any commercial or financial relationships that could be construed as a potential conflict of interest.
